# Morphological and Tribological Properties of PMMA/Halloysite Nanocomposites

**DOI:** 10.3390/polym10080816

**Published:** 2018-07-25

**Authors:** Zina Vuluga, Mihai Cosmin Corobea, Cristina Elizetxea, Mario Ordonez, Marius Ghiurea, Valentin Raditoiu, Cristian Andi Nicolae, Dorel Florea, Michaela Iorga, Raluca Somoghi, Bogdan Trica

**Affiliations:** 1 National Research and Development Institute for Chemistry and Petrochemistry-ICECHIM, Bucharest 060021, Romania; ghiurea@gmail.com (M.G.); vraditoiu@yahoo.com (V.R.); ca_nicolae@yahoo.com (C.A.N.); dflorea22@yahoo.fr (D.F.); michaelaiorga496@gmail.com (M.I.); ralucasomoghi@yahoo.com (R.S.); trica.bogdan@gmail.com (B.T.); 2 Fundacion Tecnalia Research and Innovation, Donostia-San Sebastian 20009, Spain; cristina.elizetxea@tecnalia.com; 3 Maier Technology Centre, R&D Department, Polígono industrial Arabieta 48320, Spain; marord@maier.es

**Keywords:** PMMA, halloysite nanotube, nanoscratch, friction, morphology

## Abstract

From an environmental and cost-effective perspective, a number of research challenges can be found for electronics, household, but especially in the automotive polymer parts industry. Reducing synthesis steps, parts coating and painting, or other solvent-assisted processes, have been identified as major constrains for the existing technologies. Therefore, simple polymer processing routes (mixing, extrusion, injection moulding) were used for obtaining PMMA/HNT nanocomposites. By these techniques, an automotive-grade polymethylmethacrylate (PMMA) was modified with halloysite nanotubes (HNT) and an eco-friendly additive *N*,*N*′-ethylenebis(stearamide) (EBS) to improve nanomechanical properties involved in scratch resistance, mechanical properties (balance between tensile strength and impact resistance) without diminishing other properties. The relationship between morphological/structural (XRD, TEM, FTIR) and tribological (friction) properties of PMMA nanocomposites were investigated. A synergistic effect was found between HNT and EBS in the PMMA matrix. The synergy was attained by the phase distribution resulted from the selective interaction between partners and favourable processing conditions. Modification of HNT with EBS improved the dispersion of nanoparticles in the polymer matrix by increasing their interfacial compatibility through hydrogen bonding established by amide groups with aluminol groups. The increased interfacial adhesion further improved the nanocomposite scratch resistance. The PMMA/HNT-EBS nanocomposite had a lower coefficient of friction and lower scratch penetration depth than PMMA/HNT nanocomposite.

## 1. Introduction

Over the past 10 years, consumer attraction to black and high-gloss products increased considerably. A key property that influences consumer perception of the overall quality of the product is scratch resistance, besides gloss, colour and the sensation felt when touching the surface. Scratch resistance of plastics is becoming increasingly important due to the growing use of plastics in many areas of the industry (automotive, electrics, electronics, furniture, etc.) and the trend to eliminate paint in favour of moulded-in-colour [1,2]. A product with a few visible scratches does not meet consumer expectations and is perceived as a low-value product [3]. Instead, products that do not show visible damage after use are quality products, and the consumer remains faithful to the product manufacturer in the event of a new purchase. Most of plastic materials like polyamide (PA) or styrene-based polymers (ABS, SAN) have good mechanical properties but low-gloss appearance and a limited scratch and abrasion resistance, resulting in negative aesthetic properties. For automotive parts, poly(methyl methacrylate) (PMMA) is preferred because it is transparent, has good injection moulding characteristics, chemical resistance, very good weather resistance, the pleasant acoustics, good mechanical properties and low cost [4,5]. However, non-modified PMMA behaves in a brittle manner to impact and is not very scratch-resistant [6]. To meet the requirements of the automotive industry, the surfaces of plastic parts need to be modified, either with paints, or with thin polymeric films. Although unpainted products have lower scratch properties than their counterparts [7], however, for cost reduction and compliance with environmental regulations, it is necessary to find new additives to remove the painting step and significantly and permanently improve the scratch resistance of products [8]. Organically modified polysiloxanes, slip additives (oleamide, erucamide, stearyl erucamide, etc., that migrate to the surface) and special mineral fillers are the most used categories of additives in the automotive industry to improve the scratch resistance of plastics [1]. The effectiveness of these additives depends on particle size, quantity, basic polymer matrix and processing technology and has been proved mostly for polyolefins [9,10,11,12,13]. For maximum efficiency, more than one additive of the same category or mixtures of different categories are used [8]. Often, obtaining the desired properties means a series of compromises. For example, to obtain improved scratch resistance, it is necessary to increase the tensile strength and rigidity of the polymeric composite which unfortunately affects hardness and impact resistance or induces the loss of optic and rheological properties and vice versa. Migratory additives such as erucamide, behenamide and stearamide have traditionally been used to improve scratch resistance of PMMA [14]. Fatty acid amides migrate to the surface of plastics and form a thin film, which has as effect reducing the coefficient of friction and increasing scratch resistance. However, amides provide only short-term scratch resistance and their migration influences the final appearance of the surface regarding the gloss. Nanoparticles like nanosilica, zinc oxide (ZnO), zirconium oxide (ZrO2), nanoclay, calcium carbonate, talc, kaolin or wollastonite have a significant effect on scratch performance [1,15,16,17,18]. Surface treatments with silane may improve dispersion and therefore improve scratch resistance.

To achieve large improvements in stiffness and/or strength properties, important for scratch resistance (in terms of residual scratch depth), whilst keeping as many as possible of the other properties, nanoreinforcing additives are increasingly used. Halloysite nanotube (HNT) is harmless, can be simply processed, has a high degree of structural perfection and superior mechanical properties (such as high rigidity, good tensile and flexural strength), as well as high susceptibility for dispersion in polymer matrices [19,20]. HNTs are composed of several siloxane groups, but only few hydroxyl ones, which provide the ability of material to form hydrogen bonds and thus better dispersion potential [21]. In each halloysite nanotube (HNT), the external surface is composed of siloxane (Si–O–Si) groups, and the internal surface consists of an array of aluminol (Al–OH) groups [22]. One way to improve the dispersion of HNT in a polymer matrix and obtaining nanocomposites with enhanced thermal stability and mechanical properties is the surface modification of HNT with quaternary ammonium salts [23], organosilane such as γ-aminopropyltriethoxysilane [22,24] and *N*,*N*′-Ethylenebis(stearamide) (EBS) [19,25]. EBS is a synthetic wax used as a dispersing agent or internal/external lubricant for benefits in plastic applications to facilitate and stabilize the dispersion of solid compounding materials to enhance processability, to decrease friction and abrasion of the polymer surface. The amide groups (–CONH–) from EBS can interact with the hydroxyl groups from the HNT surface, contributing also to better dispersion of nanotubes [19]. EBS was reported also to induce different structures and chain packaging in polar polymers like PLA [26].

This work refers to the characterization of PMMA/HNT and PMMA/HNT/EBS nanocomposites. Several papers already investigated some composites with PMMA matrix and HNT fillers, dealing with very particular cases (like biomaterials). Such applications included PMMA bone cement obtaining with over 5 wt % HNT (modified with gentamicine) [27]. In this case, PMMA (obtained by an initial polymerization process) mixed with modified HNT, was used to a second direct polymerization process with monomer to embed with fresh polymer and to “freeze” the material mixture. Few data were found on materials with commercial grades PMMA based on pristine HNT. Fewer data were found on automotive grades (or other thermoplastic) PMMA materials obtained by injection moulding. It is noteworthy to mention that the effects of (*co*)addition of HNT and EBS into PMMA matrix have not been reported. Furthermore, relatively very few studies were focused on nanoscratch damage in PMMA nanocomposites. Over time, to define the appropriate parameters that can be used to evaluate the scratching behaviour of amorphous polymers, the characteristic strain on PMMA during scratch tests at various temperatures [28], as well as the scratch characteristics on PMMA as a function of the concentration of a slip agent [7] have been studied.

In this paper, addition of pristine HNT (or in combination with a specific additive) can bring for PMMA-injected materials, several improved features in what concerns the materials design, properties enhancement and especially the balance between properties (needed in real applications). To the best of our knowledge, this research can be considered a first example for proving how simple polymer processing technique can be driven, to exclude supplementary synthesis processes (for filler functionalizing with organic moieties, use of solvents, purification and so on). By this approach the entire process (i.e., for an injected sample) can be managed towards favourable interface interaction between phases, in a synergistic manner for final properties design.

In this paper, the relationship between the morphology/structure (XRD, TEM, FTIR) and the conventional and nano mechanical properties of PMMA nanocomposites is discussed. Based on possible interactions between the components, an explanation of the mechanical and nanomechanical properties as well as the nanoscratch damage of PMMA/HNT nanocomposites was attempted. In dynamical conditions by the melt processing method, nanocomposites with 2 wt % HNT or HNT modified with EBS were obtained.

## 2. Materials and Methods

### 2.1. Materials

PLEXIGLAS^®^ 8N black 9V022, an amorphous thermoplastic moulding compound PMMA (automotive parts grade), was supplied by Evonik Röhm GmbH, Darmstadt, Germany. Halloysite nanotubes (HNT), natural aluminosilicate clay with a hollow tubular morphology (nanoscroll) [29], was supplied by NaturalNano Corp, Rochester, NY, USA, in white colour powder form. *N*,*N*′-ethylenebis(stearamide) (EBS), with molecular weight of 593.02 g/mol, density of 0.97 g/cm^3^ and melting range of 144–146 °C, was supplied by Sigma Aldrich, Saint Louis, MO, USA.

### 2.2. Preparation of Nanocomposites and Samples

#### 2.2.1. Modification of HNT with EBS

The surface of HNT was modified by treatment with EBS, in dynamical conditions using a Brabender Plastograph ( Brabender GmbH & Co KG, Duisburg, Germany), at 80, 120 and 160 °C, 1 h and 100 rpm. The weight ratio between HNT and EBS was 2.33: 1 (70:30). The ratio between HNT and EBS was chosen in agreement with other already existing reports like Pluta et al. [26] (described for more polar matrices like polylactic acid). Before use, the HNT was dried under vacuum for 2 h at 70 °C. The samples were denoted: (HNT-EBS)80, (HNT-EBS)120 and (HNT-EBS)160, respectively.

#### 2.2.2. Obtaining of PMMA/HNT Nanocomposites

Before use, PMMA and HNT were dried at 80 °C for 4 h and at 70 °C under vacuum for 2 h, respectively, to minimize the water content. To achieve a uniform dispersion of HNT/surface modified HNT into the polymer matrix, masterbatches based on PMMA with 20 wt % HNT/surface modified HNT have been prepared in dynamical conditions using a co-rotating twin screw extruder type DSE 20 Brabender (Brabender GmbH & Co KG, Duisburg, Germany) at a screw rate of 180 rpm. Extruder temperature profile from hopper to die was 220, 230, 240, 240, 250 and 240 °C, respectively. The extruded filaments were cooled down with air and then were granulated. The masterbatches were denoted as CPA-80, CPA-120 and CPA-160, respectively. Prior to feeding into the extruder, the granules of PMMA and masterbatches were mixed in a rotating mixer at RT for 15 min. The dilution of masterbatches with PMMA was done using the same conditions as for masterbatches obtaining. The extruded filaments were cooled down in a water bath and then were granulated with a Brabender Pelletizer, the take-off speed being 9 ± 1 m/min. A neat PMMA (denoted as PMMA) and PMMA with 0.86 wt % EBS (denoted as PMMA-EBS) were obtained in the same conditions and served as control samples. The nanocomposites with 2 wt % HNT/surface modified HNT were denoted as PMMA-HNT, PMMA-(HNT-EBS)80, PMMA-(HNT-EBS)120 and PMMA-(HNT-EBS)160, respectively. For clarity, the formulations and the processing conditions for all prepared and characterized samples are presented in Table S1.

For mechanical testing, nanocomposite granules were injection-moulded using Engel ES 40/22 injection-moulding machine, into standard test specimen for tensile, impact and wear tests. The temperature profile ranged from 220 to 240 °C and the mould temperature was kept at 80 °C.

### 2.3. Characterization

#### 2.3.1. Thermal Analysis

TGA was performed on TA-Q5000IR (TA Instruments, New Castle, DE, USA) using nitrogen as the purge gas at a flow rate of 50 mL/min. The thermograms were acquired between 25 and 700 °C at a heating rate of 10 °C/min. For each measurement, duplicate samples weighed in platinum pan, about 7–8 mg, were used.

Differential Scanning Calorimetry (DSC) measurements were performed on samples of 8–12 mg, by using a DSC Q2000 (TA Instruments, New Castle, DE, USA) under helium flow of 25 mL/min. The glass transition temperature (*T*_g_), melting temperature (*T*_m_), maximal temperature (*T*_relax_) for the enthalpic relaxation and the corresponding enthalpies were determined from the first and second heating scans as follows: equilibration at 30 °C, modulate ±0.796 °C every 30 s, isotherm at 30 °C for 3 min, heating from 30 to 160 °C at a rate of 10 °C/min^−1^, isotherm at 160 °C for 3 min, then cooling back to 25 °C, isotherm at 25 °C for 3 min and, reheating to 160 °C at a rate of 10 °C/min^−1^. PMMA/HNT samples were sealed into Tzero aluminum pans and an empty Tzero aluminum pan was used as reference.

#### 2.3.2. Structure/Morphology

##### XRD

X-ray diffraction measurements were carried out with a Rigaku Smartlab diffractometer (Rigaku Corporation, Tokyo, Japan) using a Cu Kα radiation. In this experiment, the accelerating voltage of the generator radiation was set at 45 kV and the emission current at 200 mA. The diffractograms were recorded in parallel beam geometry over 2θ = 5° to 50° continuously at a scan rate of 4°/min.

##### FTIR

FTIR spectra were recorded on a Jasco FTIR 6300 spectrometer (JASCO Int. Co., Ltd., Tokyo, Japan) equipped with a Golden Gate ATR (diamond crystal) from Specac Ltd., London, UK, in the range 400–4000 cm^−1^ (30 scans at a resolution of 4 cm^−1^).

##### TEM

Transmission electron microscopy was performed using a Tecnai™ G2 F20 TWIN Cryo-TEM, 2015-FEI Company™ equipment, Hillsboro, OR, USA. Sample evaluation was performed in classical TEM, electron diffraction and scanning transmission electron microscopy mode as well. EDX analysis was carried out in scanning transmission electron microscope (STEM) mode using an SDD Oxford Instruments X-Max detector (Oxford Instruments, Oxford, UK). HNT samples were prepared by mixing the dried samples with ethanol and then ultrasonicated for 30 min for a better dispersion. A small droplet of dispersion was poured on a holey carbon grid, without staining. Polymer-based samples were embedded in Epon Resin and sectioning small slices of samples, at room temperature, with the system Leica Ultracut EM FC7 microtome (Leica Microsystems GmbH, Wetzlar, Germany). EDX analysis allowed a proper identification of the organic phase (rich in N element for EBS, rich in Al and Si for the HNT, respectively only C and O for the polymer phase).

#### 2.3.3. Mechanical Properties

##### Conventional Tensile and Impact Testing

Tensile properties of the composites were determined according to ISO 527, with 5 mm/min for the tensile strength and 1 mm/min for the modulus of elasticity, using an Instron 3382 Universal Testing Machine (Instron Corporation, Norwood, MA, USA) and seven specimens for each test. The Charpy unnotched impact strength of the composites was determined according to ISO 179-1, using a Zwick HIT5.5 Pendulum Impact Testers (Zwick Roell AG, Ulm, Germany) and seven specimens for each test.

##### Nanomechanical Characterization

*Nanoindentation*

Nanoindentation tests have been performed at room temperature on a TI Premier system (Hysitron Inc., Minneapolis, MN, USA) using a three-side pyramidal Berkovich tip with total angles of 142.35 deg (radius of curvature of 150 nm). Four automated indentations have been performed on the sample surface, 2 horizontal and 2 vertical, with 1 μm the distance between the indents, using the trapezoidal load function. The normal load was linear-loaded during 5 s until the maximum load of 10 mN was reached. The peak load was held for 2 s in order to minimize the viscoelastic effects of the polymer during the unloading (creep or relaxation). The force was then unloaded for another 5 s. Based on this experimental set-up, the load-displacement curves were obtained. The nanomechanical properties were obtained by the values of hardness (*H*) and reduced modulus (*E*_r_), calculated using Oliver-Pharr method [30]. All values were taken as the average of 4 indentations.

*Nanoscratch*

The nanoscratch experiments have been performed using the same Hysitron TI Premier and employing the same Berkovich tip used for nanoindentation. A normal load of 5 mN was applied in a controlled manner to the indenter tip and the lateral force and lateral displacement were recorded as a function of time. In the first stage, the tip was brought in contact with the sample for indenting, during 5 s, until the specified load was attained. The peak load was held for 3 s and in the second stage the indenter started sliding on the sample surface, during 50 s. After another 3 s of force holding the tip was unloaded during 5 s. Each scratch consisted in a 20 μm scratch length and a 66 s duration. The surface topography of the samples was characterized using the scanning probe microscopy (SPM) mode of the TriboIndenter (Hysitron, Inc., Minneapolis, MN, USA). After scratching, a representative 25 μm in situ SPM image was obtained on each sample for post-test qualitative surface characterization, and for quantitative nanoscratch depth analysis (coefficient of friction, cross profile topography, residual deformation of material during the scratch, surface roughness and depth of scratch).

## 3. Results and Discussion

### 3.1. Thermal Properties

#### 3.1.1. TGA

**Figure 1:**
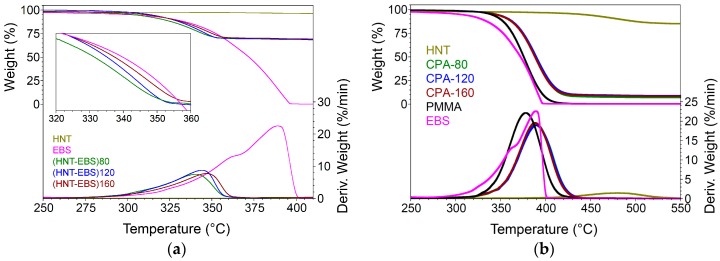
TGA results (**a**) catalytic effect of halloysite nanotubes (HNT) for *N*,*N*′-ethylenebis(stearamide) (EBS) thermal decomposition in HNT-EBS decorated hybrids (**b**) synergistic effects between EBS and PMMA decomposition fractions able to improve masterbatches maximal decomposition temperature.

**Figure 2:**
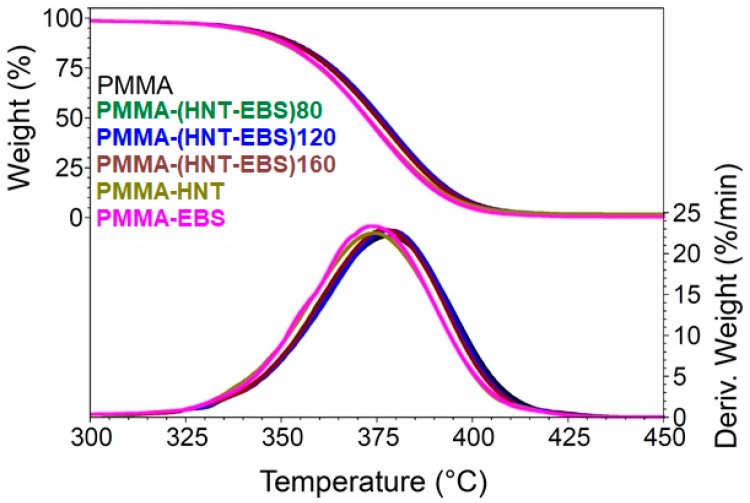
Synergistic effects between EBS and HNT, acting on PMMA decomposition fractions.

**Figure 3:**
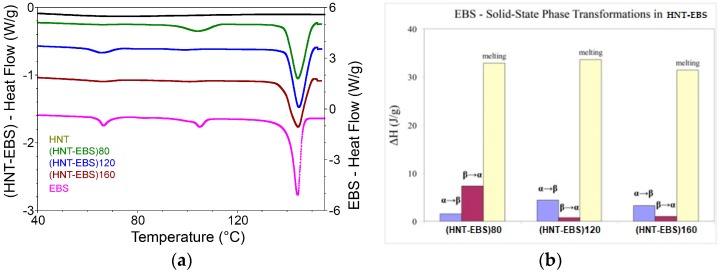
Phase transition of EBS in the HNT-EBS fillers obtained in different processing conditions compared with initial neat EBS and HNT. (**a**) DSC thermograms; (**b**) enthalpies of phase transformations (alpha-beta, beta-alpha and melting) of EBS in HNT-EBS fillers.

Thermogravimetric analysis (TGA) emphasized the structural changes for different processing stages and the reflection of these changes in the thermal properties of the composite materials (Figure 1, Figure 2 and Figure 3). Thermal properties were found in close correlation with the behaviour reflected in the nano and macro scale properties of PMMA composites. Despite the small concentration, HNT (2 wt %) modified with EBS, improved PMMA thermal stability. Like in the mechanical properties section, EBS or HNT alone are inducing lower thermal stability for the PMMA matrix but together a synergistic effect was attained. Hydrogen bonds between organic compounds and aluminol groups from alumino-silicates can lead to strong interfaces like in covalent bonding in certain cases [31]. The main vector was proven to be the alumina sites from the alumino-silicates. In our case, the charge compensation (for HNT 1:1 structure) is lower if compared with 2:1 layered structures like montmorillonite, therefore a certain increase in activity should be expected. In the case of HNT decorated with EBS, the acid sites are acting as catalyst for the EBS thermal decomposition. The stronger the interaction by hydrogen bonding was, the better the catalytic effect became. A faster decomposition and a lower thermal stability of the HNT modified with EBS at 120 °C was noticed (Figure 1a). The different degradation behaviour of the three hybrids is probably due to different forms of EBS crystallization (mainly alpha and beta phase transitions) induced by the mixing temperature [32]. Crystallization forms of EBS may favour coupling to the HNT body which will be reflected in the improvement of interface with the polymer matrix and, consequently, in the enhancement of properties. The char obtained from the organic compound then fastens the inorganic dehydroxylation stage of the aluminosilicate between 400 and 500 °C.

The TGA of the masterbatches revealed the same synergistic effect (EBS-HNT) for the PMMA, the thermal resistance of the polymer being improved following the trend of that of HNT-EBS hybrids in initial processing conditions (HNT modified with EBS at 120 °C, then HNT modified with EBS at 160 °C and then HNT modified with EBS at 80 °C) (Figure 1b). The thermolytic radical decomposition of PMMA [33] starts around 300 °C, by forming tertiary and secondary carbon radicals. These radicals are able to recombine in mirror (in the same thermal event and temperature around 300 °C) with the in situ EBS thermal scission products [34,35]. EBS decomposition occurs in the most probable position (in the beta carbon position) of the carbonyl group (lowest energy) giving one radical, then the rest of the chain remains with a reactive double bond able also to interact with PMMA radicals. Therefore, the reactivity and the reaction place provided almost immediate recombination opportunity for both partners (EBS and PMMA molecules subjected to decomposition) and the overall stabilization for degradation occurs [36]. The improvement of thermal stability in the case of masterbatches (compared with neat PMMA) was increased with almost 30 °C for the maximal decomposition temperature. The mirroring effect of the radical recombination (from EBS and PMMA) [37] can be reflected by the fact that the onset stage was less affected by the synergistic effect; meanwhile, the main event (maximal decomposition stage) was considerable affected by the radical recombination. Thermal decomposition of PMMA is described in Scheme S1: (a) homolytic bond dissociation and (b) char production. Reactions suggested for thermal degradation of EBS are presented in Scheme S2.

In the final PMMA-HNT-EBS nanocomposites with 2 wt % HNT, less than 1 wt % EBS was not enough to highlight an improvement in the overall thermal stability (Figure 2). As it can be seen, both HNT and EBS are inducing a faster degradation of the PMMA but like in the mechanical properties the combination of the two (EBS and HNT) brings a synergistic effect. EBS alone starts the degradation earlier than PMMA (Figure 1b) when PMMA radicals are not available. HNT alone (without EBS) because of a high hydroxyl content and acid alumina sites induces a faster degradation of PMMA [38,39]. The combination of EBS-HNT provides the synergistic effect and the stability of PMMA remains unchanged (in the final composites compared with neat PMMA).

#### 3.1.2. DSC

Thermal events in DSC revealed significant features of the phase distribution in the anisotropic bulk materials and the fine-tuning of the structure, in order to achieve the highlighted properties (in nano and macro scale). First, the alpha and beta phase transitions of EBS were confirmed by DSC, in good agreement with the same intervals highlighted by FTIR and XRD (Figure 3). These transitions confirmed the speculated processing approaches, drawn by the experimental set-up in Brabender mixing stage (80, 120, 160 °C for EBS-HNT). The initial EBS as received at room temperature (without further thermal modification) consists in a mixture of alpha and beta crystals, which after 60 °C starts an endothermic event specific for the alpha to beta full transition. These data were in good agreement with the ones found in literature [26]. Around 80 °C, the EBS can be found mainly in the beta form, which around 110 °C converts again in the alpha form. At 120 °C, the EBS was mainly in the alpha form again, and then at around 140 °C the complete melting of EBS begins in a classic endothermic event. In the HNT presence (HNT has no transition on the mentioned interval), the phase changes of EBS were influenced due to the specific interaction and the specific form at the processing temperature. For the sample processed in the Brabender mixer at 120 °C, EBS was coupled on HNT mainly in the alpha form. This form was also favourable for the later interaction with PMMA. At the same time, this sample had the largest amount of free EBS (again important for further interactions with PMMA).

**Figure 4:**
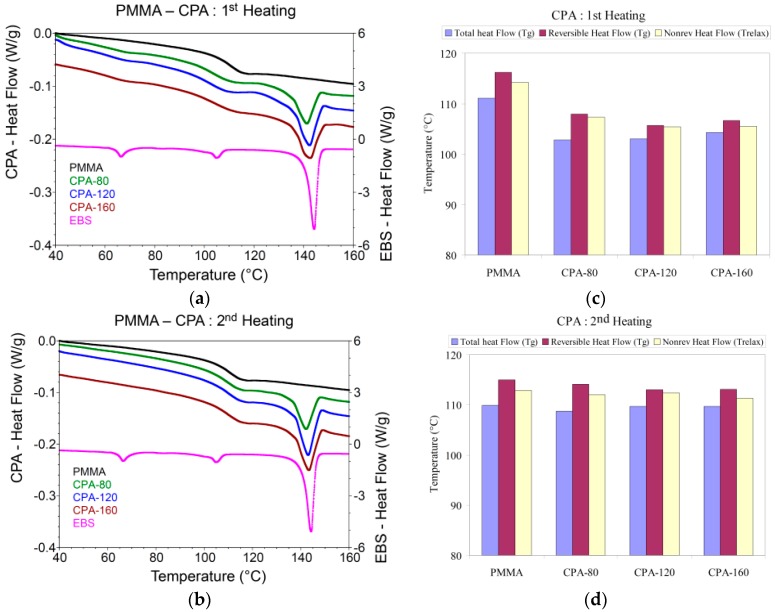
PMMA chain transition (*T*_g_, *T*_relax_) in the HNT–EBS masterbatches. DSC thermograms: (**a**) the 1st heating and (**b**) the 2nd heating). *T*_g_ (from the Total and Reversible Heat Flow) and *T*_relax_ (from the Non-Reversible Heat Flow): (**c**) the 1st heating and (**d**) the 2nd heating).

**Figure 5:**
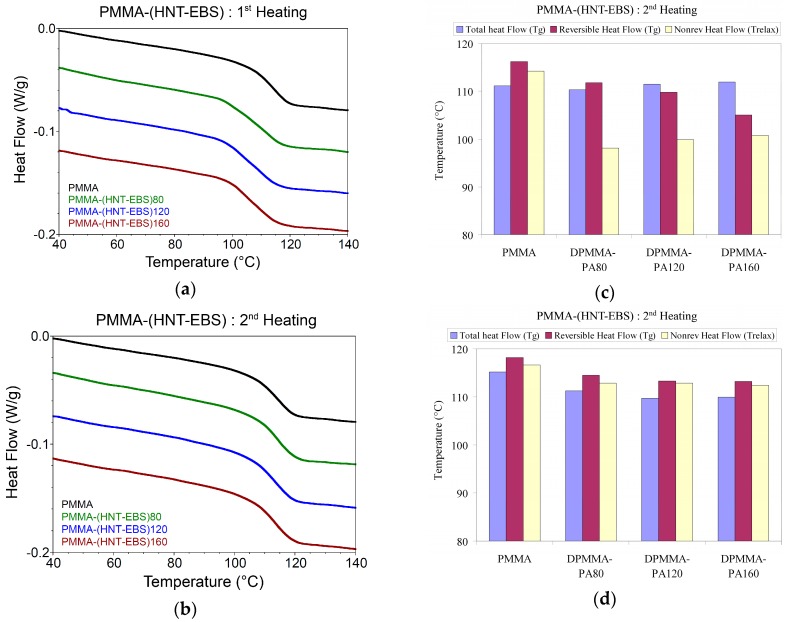
PMMA chain transition (*T*_g_, *T*_relax_) in the HNT–EBS final nanocomposites. DSC thermograms: (**a**) the 1st heating and (**b**) the 2nd heating). *T*_g_ (from the Total and Reversible Heat Flow) and *T*_relax_ (from the Non-Reversible Heat Flow): (**c**) the 1st heating and (**d**) the 2nd heating).

PMMA *T*_g_ and *T*_relax_ (Figure 4 and Figure 5) were affected by the processing condition of the filler and the plasticizing effect brought by free EBS. This phenomenon was expected and reflected also on the relaxation chain temperatures (*T*_relax_) level. *T*_relax_ were decreased in the presence of HNT-EBS, all thermal events indicating the improvement of processing ability of PMMA materials next to the properties enhancement.

The effect seen in masterbatches (high filler content) in DSC analyses was preserved also in the final nanocomposites (less than 2 wt % filler). The results were in good agreement with elastic and plastic behaviour seen in mechanical properties (tensile, axial strain, impact and modulus) reflected in DSC by the small modifications of the *T*_g_ and *T*_relax_ of the PMMA in the presence of HNT-EBS.

### 3.2. Structure/Morphology

#### 3.2.1. XRD

**Figure 6:**
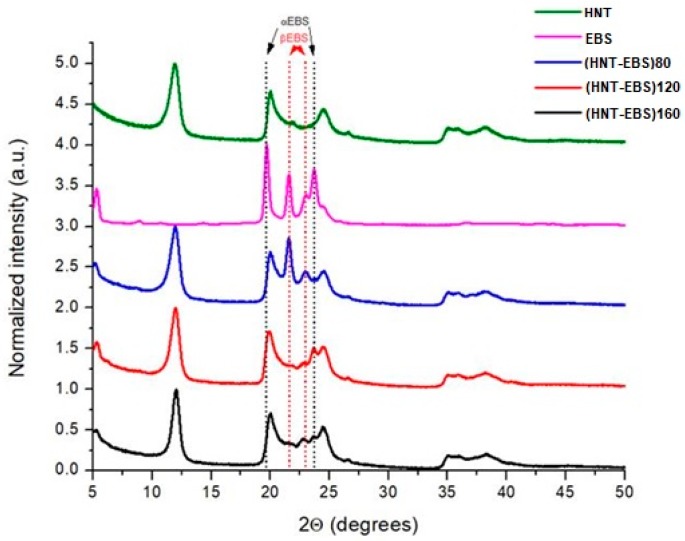
Structures of the EBS-HNT hybrids obtained at different temperature (80, 120, 160 °C). Free EBS occurrence in different crystallizing forms in HNT modified with EBS.

**Figure 7:**
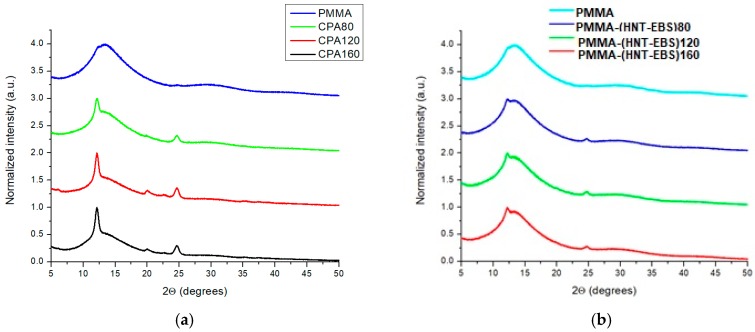
XRD patterns for the masterbatches (**a**) and final PMMA nanocomposites (**b**).

Diffraction patterns XRD for the HNT (Figure 6) revealed that the interplanar distance for the nanoscroll wall structure at around 7.4 Å. EBS crystalline structures were also revealed at room temperature with the coexistence of the alpha and beta form of crystallization. In the presence of EBS, HNT wall seemed very little affected with differences of less than 0.3 Å, indicating that EBS penetration inside the HNT tube (for the inner aluminol groups) was a very improbable event. In this later context, a partial debonding of the exterior HNT sheet (scrolled layer) should not be excluded. For the PMMA masterbatches and final composites, the presence of all partners was confirmed even at low concentration (2 wt % HNT), confirming by this way the relation between structure and the induced properties (Figure 7).

**Scheme 1:**
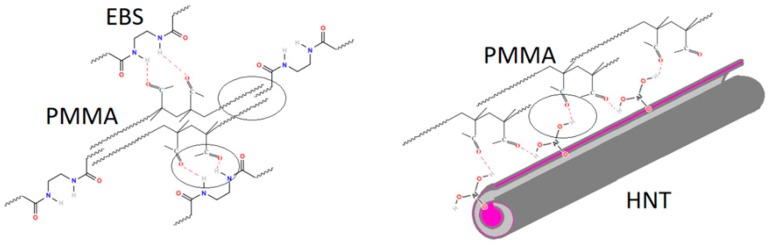
Idealized structures of the most probable interactions available between partners (competitiveness of the PMMA-EBS vs. PMMA-HNT).

For the different modification of HNT with EBS correlated with the nano and macro scale mechanical properties, it seems that alpha form of EBS was able to promote the best interaction in both masterbatches and final nanocomposites in relation with the PMMA matrix (from Scheme 1). The correlation between tensile strength and the occurrence of the alpha/beta form of EBS for the initial HNT treatment reflects the tuning possibilities given by the processing route for the final material properties. Like shape memory, even if the subsequent processing temperatures highly exceed the EBS melting point, the induced properties can be controlled.

**Scheme 2:**
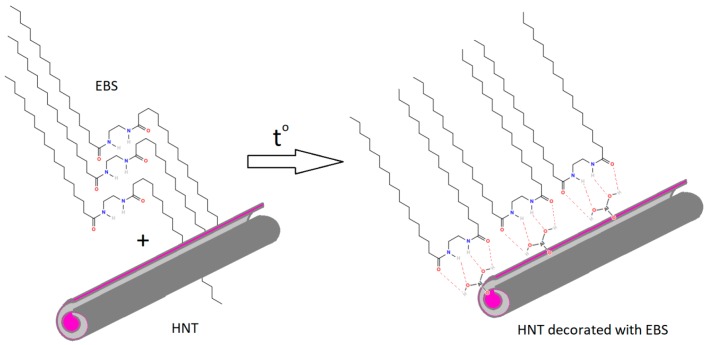
Idealized structures with the strongest interaction available between EBS and HNT (and the most probable based on sites reactivity aluminol vs. silanol).

XRD results also showed an increase in HNT crystallites dimensions, confirming indirectly the interactions between aluminol sites and amide groups (from Scheme 2). The decoration of HNT with EBS was also confirmed by the TEM details reflecting EBS crystals on HNT surface. In the case of PMMA final nanocomposites and their initial masterbatches, the crystallite dimensions were expected to increase even more, but the peak overlapping and the complex packaging in the amorphous region made the assessment very difficult and the interpretation too subjective.

#### 3.2.2. FTIR

**Figure 8:**
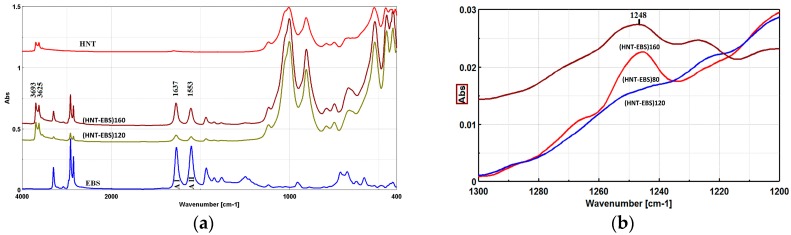
FTIR-ATR spectra of (**a**) HNT-EBS composites; (**b**) band at “1248 cm^−1^”.

EBS interacts through hydrogen bonds established by amide groups with HNT aluminol residues, as can be observed in Figure 8. The amide band I (A I) corresponds to the stretching vibration of the carbonyl group coupled with the in-plane bending of the amino group and the stretch of the C–N bond. Amide II (A II) originates in the bending vibration of the N–H bond and stretching of the C–N group in equal proportion. The intensities of the amide absorption bands I and II are sensitive to environmental conditions and vary because of the hydrogen bonding of the carbonyl and amino moieties. Therefore, the ratio was attributed to differences in secondary and possible tertiary structure of EBS (similar H–bonds like in proteins) [40].

Analysing the ratios between the amide I and II band intensities of EBS and HNT-EBS, there was a decrease in the amide band II intensity at 1554 cm^−1^ with respect to amide I at 1636 cm^−1^, due to associations through hydrogen bonds involving the N–H groups, as it can be seen in Figure 8a.

In the case of HNT, there are two sharp bands at 3693 and 3625 cm^−1^, respectively. The first is due to the stretching vibration of the inner surface OH bonds, and the second to the inner OH groups near the aluminium atoms. Decreasing the intensity of the second band in the case of composites indicates that these groups are involved in hydrogen bonding with EBS amide groups.

Regarding inter-conversion of crystalline forms of EBS during the thermal treatment, as was already reported [32] three bands are characteristic in the FTIR spectrum, situated at 1248, 955 and 940 cm^−1^. The presence of the band at 955 cm^−1^ in the FTIR spectrum and the absence of the other two bands is characteristic to the alpha crystalline form, while the absence of the band at 955 cm^−1^ and the appearance of the other two bands is characteristic to the beta form. Analysis of the HNT-EBS composites shows the disappearance of the peak at 1248 cm^−1^ when they are prepared at 120 °C, which demonstrates the presence of the alpha crystalline form in these composites, as it can be observed from Figure 8b. Unfortunately, in the range 940–960 cm^−1^ the characteristic bands of the crystalline forms cannot be identified due to the superimposing with the existent HNT bands.

Considering the molecular structures of the partner involved (PMMA matrix, HNT-filler and EBS) and XRD and FTIR results, several combinations of interactions can occur during the processing stage. These interactions have different energies and formation probabilities (when competing in the same space) [41,42]. The steric hindrances are playing also an important role for molecules confinement in the composite structure. Furthermore, these structures can be reflected indirectly by the associated interface and the induced properties of the final materials (Scheme 1 and Scheme 2).

#### 3.2.3. TEM

**Figure 9:**
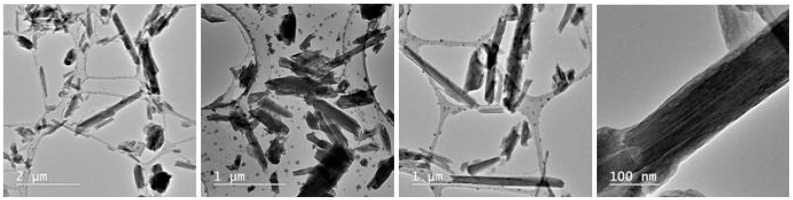
TEM images for HNT particles before modification at different magnifications.

**Figure 10:**
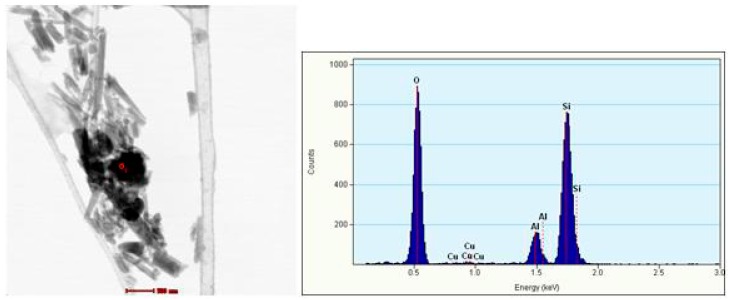
TEM-STEM (bright field) imaging and EDX data for HNT phase identification.

TEM results reflected the HNT morphology induced by the tubular structure and the changes during the processing trials. Initial HNT, a natural grade, had a relatively broad dimension distribution, with length starting from 60–100 nm up to 1.5–2 microns. The exterior diameter starts from 50 up to 200 nm (Figure 9 and Figure 10). The obtained results are in good agreement with the one already reported in literature [41].

**Figure 11:**
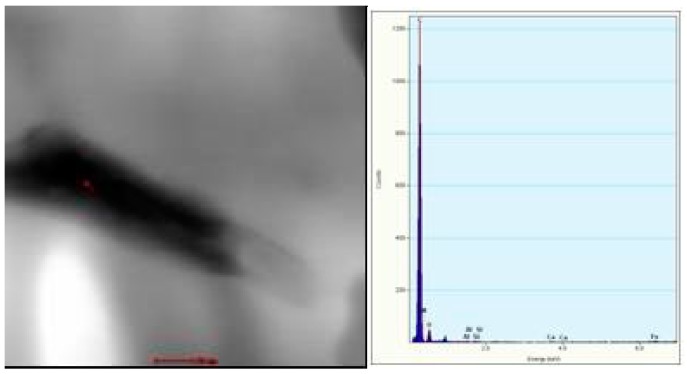
TEM–STEM details of EBS crystallized on HNT structure (sample (HNT-EBS)120).

After HNT modification at 120 °C with EBS, the crystals of EBS were identified on HNT structure. These data confirmed the crystallites increase observed in XRD after modification (Figure 11).

**Figure 12:**
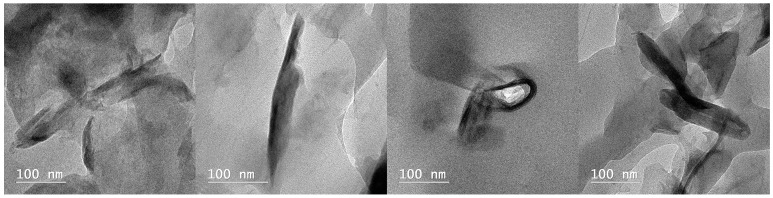
TEM images for HNT in PMMA materials obtained by favouring the interaction of EBS in the alpha form.

In TEM details for the final PMMA composites (Figure 12), HNT appear well dispersed in the polymer matrix highlighting the interface assistance promoted by EBS. The assumption involving the superficial delaminating of a small part of the sheet forming the HNT nanoscroll was confirmed by TEM details. The interaction between EBS and HNT (then EBS-PMMA) was strong enough during melt processing and high shear process (like extrusion) to promote such a debonding and, even more, some of the HNT nanotubes to be found in smaller dimension (induced by local cracking after processing condition). At this point, if we refer again to the mechanical properties (at nano and macro scale) we can observe the force of the interface interaction, since many HNT were found in a damaged state and therefore a strong decrease in mechanical properties was to be expected.

TEM and XRD confirmed the structures interaction and explained how the synergistic effect between HNT and EBS can contribute to PMMA modification. All results were in good agreement with the properties improvement at nano and macro scale.

### 3.3. Mechanical Properties

**Figure 13:**
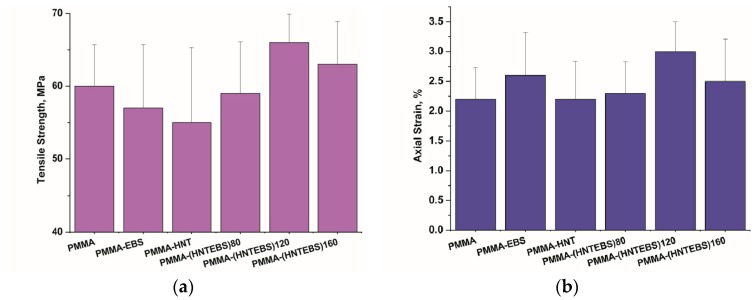
Tensile strength (**a**) and axial strain (**b**) of PMMA/HNT nanocomposites compared to PMMA and PMMA with EBS or HNT.

Mechanical properties initially showed some unexpected results involving PMMA materials with HNT filler. Despite HNT high modulus (theoretical value-140 GPa (230–340 GPa)) [43], PMMA HNT nanocomposite material showed a decrease in tensile strength (with almost 10%) when compared with neat PMMA (Figure 13a). Another result (more expected) was registered for PMMA-EBS blend, highlighting the polymer matrix weakening, by the tensile strength decrease. Yet, at a closer inspection, when only the small molecular weight additive (EBS) was used, the decrease in mechanical properties seems to be limited, when compared with PMMA samples obtained with HNT only. Up to this point, it seems that PMMA matrix was able to interact more favourably with EBS than with HNT (the later offering a weak interaction with the polymer matrix—only one level of interaction between negatively charged carbonyl, and edge aluminium sites positively charged). Moreover, HNT was expected to be found in lower dispersion degree (in comparison with other samples) and the eventual aggregates to act as stress concentrators in polymer matrix [44]. The results were also confirmed by the TEM data (presented in this study). After this first model of the filler-matrix interaction, other paths were speculated by using the HNT treated with EBS. This treatment had to cover two directions: one to promote the energy needed for the EBS molecule conformation for coupling on the HNT surface (polar amide segment orientation in the opposite direction of the hydrophobic-stearyl tails, to reduce the steric hindrance) (Scheme 2) and second, a favourable crystallization for the EBS molecules able to assemble on the interface. Overall mechanical properties highlighted the distinct behaviour induced by the two major forms (α and β) for EBS crystallizing [32]. Therefore, the different forms of EBS crystallization should be considered the main actors for the PMMA-HNT interface, since EBS can provide: (i) the polar-polar interface with HNT; (ii) hydrophobic interaction between stearyl and PMMA main chain and (iii) amide-carbonyl interaction from free EBS molecules (uncoupled on the HNT surface). The later described form of EBS (iii) can provide also the coupling with another EBS molecule confined on HNT surface (by hydrophobic chain interaction, stearyl-stearyl) for inducing different packaging of hydrophobic chains (segment) of PMMA.

The tensile strength of the final nanocomposites increases for all three types of initial thermal treatment of the HNT filler with EBS when compared with PMMA-EBS and PMMA-HNT. This improvement confirmed the structural interaction between partners on the interface based on the theoretical molecular dynamics. The improvements should be regarded more in contrast with PMMA-EBS and PMMA-HNT (and less with PMMA alone which possesses a lower properties profile i.e., scratch, hardness in the nanomechanical section). Therefore, the balance between properties was easier to establish by use of both EBS and HNT. The synergistic interactions were preserved up to the final PMMA nanocomposites and depend on EBS alpha form available for the initial HNT modification (see (PMMA-HNT-EBS)120, 160, 80 from Figure 13 and EBS alpha/beta content Figure 3).

The plastic deformation of PMMA (Figure 13b) was also influenced by the complex interface provided by EBS confined on HNT; free EBS in different crystallizing states and PMMA interaction with the two states of EBS crystals. In the case of PMMA with neat HNT, a decrease in axial strain was expected, since inorganic fillers increase in general the stiffness of the final material [45,46]. However, the axial strain was kept almost unchanged when compared with PMMA matrix. This interesting phenomenon can be explained by two directions: one by the fact that weak PMMA-HNT interactions (weaker than in PMMA-PMMA) worked in tandem with the contribution of stiffness brought by HNT nanotubes (with high modulus compared with PMMA) and second, by the recent advances in HNT research which proved the extreme flexibility of the nanotubes (by real experimental technique, not by simulations) [47]. This later flexibility of the HNT should be seriously considered also for axial strain and other elasticity-driven properties or phenomena. Lu et al. [47] proved by experimental bending of single HNT nanotubes that bends up to almost 90° were possible without damage.

An important increase in axial strain was observed for the materials which used the HNT modification with EBS at 120 °C. This increase could be assigned to the contribution of both interface elasticity (PMMA chains confined on the EBS crystals) (see also PMMA-EBS sample highlighting the same behaviour) and some contribution of the HNT nanotubes elasticity. This behaviour should be defended also by other mechanical properties, but only to a certain extent, since the interface itself can overtake loads on both rigid and elastic segments (and when break occurs, the weakest bonds will fail). The synergistic interactions observed in the tensile strength section were in good agreement with ones from axial strain. In this section also, the PMMA preference to assemble on EBS rather than on HNT surface was confirmed again.

**Figure 14:**
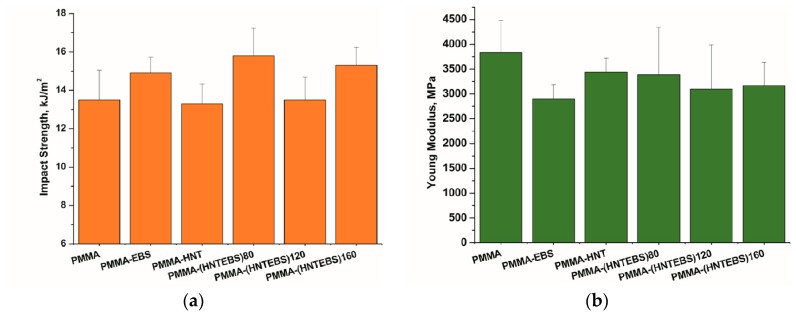
Impact strength (**a**) and Young modulus (**b**) of PMMA/HNT nanocomposites compared to PMMA and PMMA with EBS or HNT.

The impact properties (Figure 14a) account more about the behaviour and nature of the complex interface generated by HNT, EBS decorated on HNT, free EBS, different EBS conformation and PMMA chains (generating both elastic and rigid domains acting together on different mechanical loads). Usually in polymer materials, improving one mechanical property (like tensile strength for example) means the decreasing of another one (like impact strength). However, in this case, unexpectedly the impact properties were either kept constant with respect to neat PMMA matrix either increased, based probably on the previously observed contribution of the elastic domains. This kind of behaviour was generally observed for the blends or alloys of thermoplastics with thermoplastic elastomers [48,49] and their composites with silica or layered silicates. Even without thermoplastic elastomers in the case of PMMA-HNT-EBS nanocomposites, the occurrence of both elastic (EBS-HNT phase, EBS-PMMA phase) and pseudo-rigid domains (HNT agglomerates, different EBS crystals, small EBS crystals aggregates) can be found. In good agreement with axial strain, PMMA-EBS interaction plays an important role to shock overtaking and improves impact strength by a discrete elastic behaviour. The energy at break (not presented here) follows as well the same observed tendencies and correlations.

The macro scale modulus (Young Modulus obtained by mechanical analysis) follows the impact and tensile strength behaviour but not in a simple expected correlation (as expected for a fair isotropic polymer material). In our case, the access to the modulus of the PMMA matrix or HNT was very difficult, given the strong anisotropic structure and the modification of the structures during processing. In fact, the data reflected an overall modulus, as sum value of all interface areas. The small decrease in modulus should be considered also in the context of: high share induced by HNT during processing (two extrusion process with high shear, able to influence the involved structures); EBS acts not only as slip agent, but also as a bridge agent for new interactions with PMMA; EBS-PMMA interaction brings also more isotropy in the bulk composite material and provides the elasticity phases on the interface, seen also in the rest of the mechanical properties. Again, PMMA-EBS seems to be the driving force for the discrete elastic behaviour (which can be amplified by different EBS crystallization next to HNT and PMMA in the masterbatch used for the final nanocomposites).

**Figure 15:**
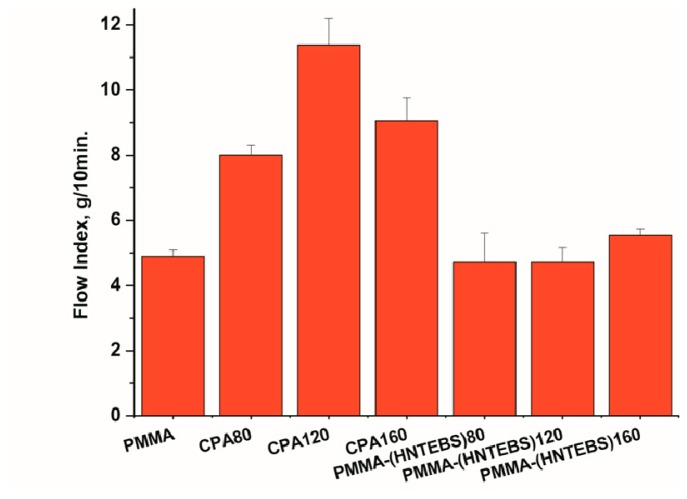
Improvements of PMMA melt flowability by the presence of HNT decorated with EBS at different temperature (80, 120, 160 °C for both masterbatches-CPA and final PMMA nanocomposites).

EBS-HNT and EBS-PMMA interactions were the driving forces not only in the final bulk materials, but also in molten state. The synergistic effect was present and the processability of both masterbatches and final nanocomposites (in the diluted form) were improved compared with neat PMMA. These results should be considered very relevant in the context of processing parameters improvement, energy consumption, and ability of melt composition to incorporate inorganic filler. Since this study assumed a PMMA grade for automotive applications, the flow index improvement will have several benefits also for the in-mould flow, allowing a good fill, especially for complex geometries or quality aspect parts. Back to each partner structure interaction, again the best results (like in tensile section) were attained when 120 °C temperature was used for HNT decoration with EBS in the Brabender mixer (Figure 15). Surprisingly, again the same improvements in properties were linked on the EBS confinement and probably induced crystallization on the HNT surface initially and in the PMMA masterbatch.

### 3.4. Nanomechanical Properties

#### 3.4.1. Nanoindentation Test Results

**Figure 16:**
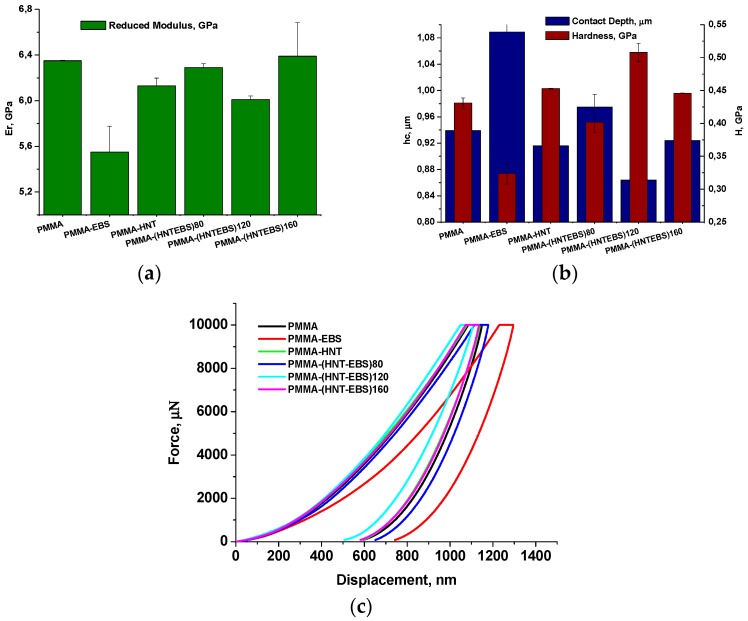
Comparison of (**a**) reduced modulus; (**b**) contact depth and hardness; (**c**) load–displacement plots of PMMA and PMMA/HNT nanocomposites.

The variation of Er and H for PMMA nanocomposites with 2 wt % unmodified HNT or EBS surface modified HNT is shown in Figure 16a.

The addition of only 0.86 wt % EBS in PMMA led to a decrease of both *E*_r_ and H, compared to neat PMMA. Thus, the *E*_r_ and *H* values (lower for PMMA-EBS), 5.55 and 0.408 GPa, compared to neat PMMA, 6.35 and 0.431 GPa, prove the lubricating effect of EBS. EBS possesses two large hydrophobic stearyl segments able to promote similar effects like the related fatty acid (well known as dispersing agents or lubricants to enhance processability of polymers) [50]. These results for EBS in PMMA were promising and could recommend EBS, also in other application for PMMA formulation. The addition of 2 wt % unmodified HNT in PMMA resulted in an increase of hardness (approximately 5% compared to neat PMMA) and the almost unchanged modulus (a decrease of approximately 3.5% may be considered within the limits of experimental error). Similar results were also obtained by other researchers. For example, K. Pal [6] reported an improved dispersion of 3 wt % HNT in PMMA matrix and, in consequence, an increasing in mechanical properties (an increase of modulus with approximately 14%, compared to PMMA). Surface modification of HNT with EBS is reflected in increasing, more or less, of *E*_r_ and *H*, respectively. It can be noticed a variation of properties depending on the technology of obtaining the nanocomposites, more precisely on how strong the interaction is between HNT and EBS and the interface adhesion with PMMA. From the three samples which contain HNT modified with EBS, the sample PMMA-(HNT-EBS)120 presented the smaller value for *E*_r_ but the highest value for *H*. These values correlate well with the values obtained for Young modulus, Charpy impact strength and Tensile strength (Figure 13a and Figure 14a). The sample PMMA-(HNT-EBS)160 presented the highest value for *E*_r_. The values obtained for hardness correlate quite well with contact depth. The lower the hardness of the sample, the greater the contact depth and vice versa (Figure 16b). Figure 16c presents load versus indentation displacement at peak loads of 10 mN for each of the samples. It can be noticed that the ”softest” sample is PMMA-EBS, with a peak depth of almost 1295 nm, while the ”hardest” is PMMA-(HNT-EBS)120, which was penetrated to a depth of about 1107 nm. However, PMMA-(HNT-EBS)120 showed the highest value for tensile strain (Figure 13b) and the highest degree of elastic recovery during unloading while PMMA-EBS showed the lowest degree of elastic recovery during unloading (Table 1).

#### 3.4.2. Nanoscratch Test Results

**Figure 17:**
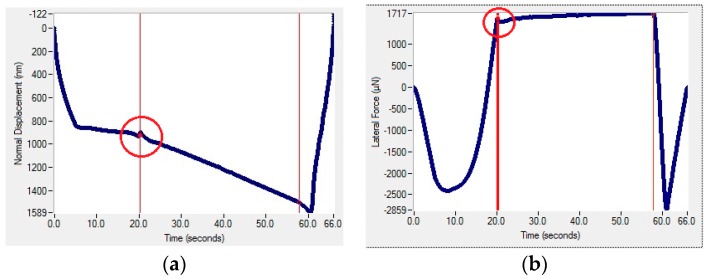
Representative plots of normal displacement (**a**) and lateral force (**b**) versus time from a 5000 μN ramping force nanoscratch test on PMMA. Critical events in the data (showing point of distinct material piled up) are circled and correspond to *h*_c_ and *L*_c_.

**Figure 18:**
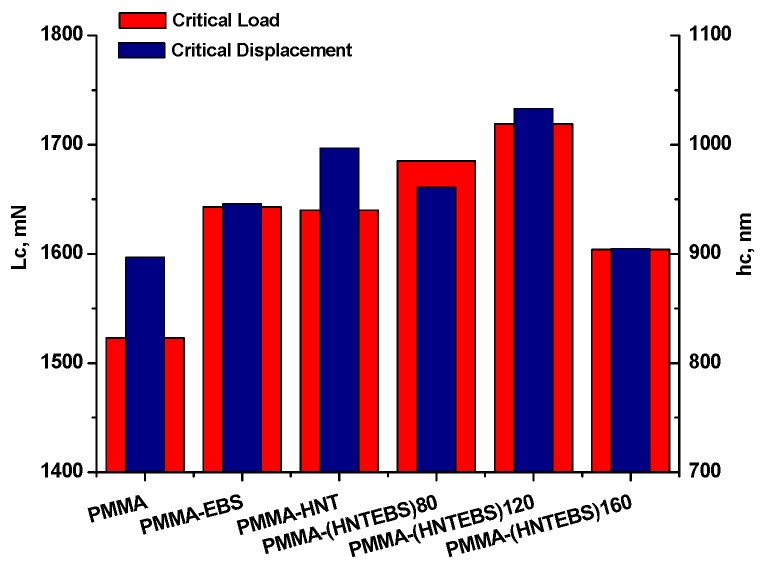
Plots of *L*_c_ and *h*_c_ data from 5000 μN ramping force nanoscratch tests on PMMA and PMMA/HNT nanocomposites.

Nanoscratch provides the capability to investigate modes of deformation and fracture of samples. Load-displacement plots, generated automatically using TriboScan software (Figure 17), can provide more information about structure/morphology and interface characteristics than a load–displacement plot obtained from an indentation test. From this data, useful information such as critical load, *L*_c_ (the load at which change in a displacement curve occurs) and critical displacement, *h*_c_, can be measured. Usually, samples with the highest *L*_c_ and *h*_c_ are be considered to have optimal scratch-resistance properties. Figure 18 shows plots of *L*_c_ and *h*_c_ data from 5000 μN ramping force nanoscratch tests performed on all samples. From this figure, it can be noticed the highest values for PMMA-HNT-EBS)120 and the lowest ones for PMMA. A representative 25 μm, 2-D topographical in situ SPM image obtained on all samples after a 5000 μN ramping force nanoscratch test is shown in Figures S1 and S2. These images, including 3-D images, were also used to calculate surface roughness, residual deformation of material during the scratch and depth of scratch for each sample. The quantitative data, *L*_c_ and *h*_c_ corroborated with post-test in situ SPM images provides additional information for characterization, including the ability to confirm critical event identification.

Looking at these images, it seems that all samples have similar topographical features of the nanoscratch. In fact, there is a clear distinction in the scratch behaviour with different microstructures. It can be noticed formation of three new surfaces by the materials piled up during nanoscratching: at the rear end of the tip, on both sides of the scratch and in front of the tip. Depending on the resistance properties of sample as well as the composition and technology employed for obtaining of sample, there are different residual widths (side pile-up) of scratch and different scratch depths. The roughness of the surface influences the lateral force/frictional force measurements and, therefore, the value of coefficient of friction. The RMS roughness (root mean square roughness, *R*_q_), the coefficient of friction (μ), the scratch depth (SD) and the scratch pile-up (rear, side and front) measured for all samples are presented in Table 2.

As observed from Table 2 there is a very good correlation among the roughness of surface, the coefficient of friction and the scratch penetration depth. The highest values for surface roughness, coefficient of friction and scratch penetration were obtained for PMMA and the lowest ones for PMMA-(HNT-EBS)160 nanocomposite. However, PMMA sample presented the lowest total pile-up of material (659 nm) compared to the highest presented by PMMA-(HNT-EBS)160 (1100 nm), given that both samples have the highest values for *E*_r_ and *H*, 6.35 and 0.431 GPa for PMMA and 6.39 and 0.446 GPa, respectively for PMMA-(HNT-EBS)160 (Figure 16a,b). In the case of PMMA, it is rather a sink-in of plastically torn material on both sides of the scratch. The side surfaces showed some ”undulations” as scratching depth increased and even a distinct material piled up (Figure S1a). This behaviour of PMMA, noticed also by G. Mallikarjunachari and P. Ghosh [51], is mainly due to its high stiffness, 3838 MPa (Figure 14b). In PMMA-EBS, which presented the lowest stiffness, 2896 MPa (Figure S1b), the presence of EBS is reflected in decreasing in surface roughness, coefficient of friction and scratch depth (174, 0.31 and 345 respectively, compared to PMMA, 432, 0.34 and 486, respectively). The EBS contribution to reducing friction and abrasion is well known. It was reported elsewhere that by using fatty acid amides up to 0.1 wt % a decreasing of friction coefficient of PMMA can be obtained, the bulk properties of PMMA being almost unaffected [14]. The scratch depth decreased in time and also the frontal material piled up decreased (75 nm for PMMA-EBS, compared to 188 for PMMA). Compared to PMMA, the presence of HNT in PMMA-HNT sample, reduced the coefficient of friction to 0.32, from 0.34 in PMMA. The reduction of the friction coefficient generated a more elastic contact and a higher amount of elastic recovered at the rear end of the tip (126 compared to 196 nm for PMMA). The lateral and frontal amount of material piled up was higher compared to PMMA. Furthermore, it can be noticed a strain in the material, like a “splint” at the end of lateral displacement of tip moving across the sample (Figure S1c). A possible explanation of this behaviour may be a weak interaction between PMMA matrix and the stiff phase. The samples which contain HNT modified with EBS behaved differently depending, on one hand, on the interaction between HNT and EBS and, on the other hand, on the interaction of PMMA with both HNT and EBS. The behaviour of sample which contains HNT modified with EBS at 80 °C is closer to that of PMMA (high values for surface roughness, coefficient of friction and scratch depth). Just like PMMA, this sample had high *E*_r_, *H* and Young Modulus but low tensile strain and therefore a low amount of elastic recovery (Figure S2a). Compared to PMMA, the scratching depth for PMMA-(HNT-EBS)80 sample was constant in time. The PMMA-(HNT-EBS)120 sample had the highest value for axial strain. This value was reflected in a reduction of the friction coefficient and a slight increase of the amount of elastic recovery frontal the tip (Figure S2b). The behaviour of sample which contains HNT modified with EBS at 160 °C (PMMA-(HNT-EBS)160) (Figure S2c) is closer to that which contains only EBS (PMMA-EBS). Both samples presented low values for surface roughness, coefficient of friction and scratch depth and the highest amount of elastic recovery frontal the tip (75 nm for PMMA-EBS and 50 nm for PMMA-(HNT-EBS)160). From these results, it may be concluded that the behaviour to scratching of PMMA/HNT nanocomposites depends on many factors that should be considered in relation with each other (structure/morphology, bulk mechanical resistance and surface properties). For this reason, it is necessary to analyze in depth all these factors to better understand each one’s contribution to increasing the scratch resistance.

## 4. Conclusions

HNTs were proven as viable fillers base for automotive grade PMMA and the related nanocomposites. The use of HNT in PMMA matrix can be seen as an alternative choice for greener nanotubes, interaction with eco-friendly additives like EBS, for automotive (polymer) materials. Future research in this direction could be interesting for reducing the use of carbon black, glass fibres and so on. All results were found in close correlations, highlighting the strong influence of the conditions used for the material processing. Using a simple processing route (mixing, followed by extrusion and injection moulding) the materials properties and features can be tuned on both nano and macro scale. EBS was proven as a promising additive also for PMMA. EBS plays a complex role for the material anisotropic nature. EBS molecules were more competitive in establishing bidentate hydrogen bridge interaction on HNT than PMMA macromolecule; were able to decorate HNT surface and even to crystallize on the nanotubes; were able to interact further with PMMA macromolecules (by both functional groups of PMMA and the hydrophobic chains) establishing rigid and flexible domains reflected in the mechanical properties; were able to influence the PMMA flow and processing ability. HNT and EBS can produce a synergistic effect reflected in mechanical and thermal properties (for PMMA nanocomposites). The initial processing route temperature of HNT with EBS can tune the masterbatches and final PMMA nanocomposites properties. This was possible by the different temperatures used to influence the EBS crystallizing (in alpha or beta form). The most influential on properties was found to be the EBS alpha form. The synergistic effect was valued also in the nanomechanical properties, found in good agreement with the macro scale ones. Nanomechanical analysis also highlighted the different contributions of the complex interface able to promote both rigid and flexible domains in the anisotropic material. The improvements obtained in nanoscratch, friction and indentation behaviour for PMMA-HNT-EBS nanocomposites (in comparison with neat PMMA—automotive grade), recommend this new class of nanocomposites and the associated technology (simple regular route for polymer processing) as an alternative material for further developments in the automotive industry. Therefore, future research should also consider other concentrations and combinations with other fillers to achieve even more progress over the properties. We used this work to demonstrate some first possibilities: without chemical functionalizing of HNT through solvent or synthesis routes; using simple polymer processing technology; for the low filler content (2 wt % HNT) able to induce properties in PMMA nanocomposites; to improve scratch behaviour (using two agents: one hardening HNT and a slip agent like EBS) especially without negative effect on the rest of the properties—important for polymer materials used in the automotive parts industry.

## Figures and Tables

**Table 1:** The residual depth after final unloading (final depth) for PMMA and PMMA/HNT nanocomposites.

Sample	PMMA	PMMA-EBS	PMMA-HNT	PMMA-(HNT-EBS)80	PMMA-(HNT-EBS)120	PMMA-(HNT-EBS)160
**hf, nm**	555 ± 6	552 ± 1	550 ± 2	526 ± 8	600 ± 3	553 ± 5

**Table 2:** The root mean square (RMS) roughness, the coefficient of friction, the scratch penetration depth and the scratch pile-up measured for all samples.

Sample	*R*_q_ (nm)	μ	SD (nm)	Rear Pile-Up (nm)	Side Pile-Up (nm)	Front Pile-Up (nm)
PMMA	432 ± 0.2	0.34 ± 0.01	486 ± 5	196 ± 5	275 ± 5	188 ± 5
PMMA-EBS	174 ± 0.02	0.31 ± 0.005	345 ± 5	320 ± 5	430 ± 5	75 ± 5
PMMA-HNT	193 ± 0.04	0.32 ± 0.005	395 ± 5	126 ± 5	390 ± 5	260 ± 5
PMMA-(HNT-EBS)80	234 ± 0.02	0.33 ± 0.001	465 ± 5	228 ± 5	450 ± 5	200 ± 5
PMMA-(HNT-EBS)120	194 ± 0.09	0.32 ± 0.001	365 ± 5	300 ± 5	450 ± 5	100 ± 5
PMMA-(HNT-EBS)160	148 ± 0.09	0.30 ± 0.01	320 ± 5	400 ± 5	650 ± 5	50 ± 5

## Supplementary Materials

The following are available online at http://www.mdpi.com/2073-4360/10/8/816/s1. Table S1: Formulations and processing conditions of all prepared and characterized samples, Scheme S1: Thermal decomposition of PMMA: (a) homolytic bond dissociation and (b) char production, Scheme S2: Reactions suggested for thermal degradation of EBS—scission in the β position of the carbonyl group (able to generate species to react with PMMA degradation species), Figure S1: 2-D and 3-D topographical in situ SPM images obtained on: (a) PMMA; (b) PMMA-EBS; (c) PMMA-HNT, Figure S2: 2-D and 3-D topographical in situ SPM images obtained on: (a) PMMA-(HNT-EBS)80; (b) PMMA-(HNT-EBS)120; (c) PMMA-(HNT-EBS)160.
