# Supplementary material for: Morphological and Tribological Properties of PMMA/Halloysite Nanocomposites
Source: Polymers (Basel). 2018 Jul 25;10(8):816. doi: 10.3390/polym10080816 (PMC6403831; doi:10.3390/polym10080816)
Supplement: Supplementary file 1 [file polymers-10-00816-s001.doc]

Morphological and Tribological Properties of PMMA/Halloysite Nanocomposites

SUPPLEMENTARY MATERIALS

Zina Vuluga^1^,*, Mihai Cosmin Corobea^1^,*, Cristina Elizetxea^2^, Mario Ordonez^3^, Marius Ghiurea^1^, Valentin Raditoiu^1^, Cristian Andi Nicolae^1^, Dorel Florea^1^, Michaela Iorga^1^, Raluca Somoghi^1^ and Bogdan Trica^1^

^1^ National Research and Development Institute for Chemistry and Petrochemistry-ICECHIM, Bucharest, Romania; [zvuluga@yahoo.com](mailto:zvuluga@yahoo.com) (Z.V.); [mcorobea@yahoo.com](mailto:mcorobea@yahoo.com) (M.C.C.); [ghiurea@gmail.com](mailto:ghiurea@gmail.com) (M.G.); [vraditoiu@yahoo.com](mailto:vraditoiu@yahoo.com) (V.R.); [ca_nicolae@yahoo.com](mailto:ca_nicolae@yahoo.com) (C.A.N.); [dflorea22@yahoo.fr](mailto:dflorea22@yahoo.fr) (D.F.); [michaelaiorga496@gmail.com](mailto:michaelaiorga496@gmail.com) (M.I.); [ralucasomoghi@yahoo.com](mailto:ralucasomoghi@yahoo.com) (R.S.); [trica.bogdan@gmail.com](mailto:trica.bogdan@gmail.com) (B.T.)

^2^ Fundacion Tecnalia Research and Innovation, Donostia-San Sebastian, Spain; [cristina.elizetxea@tecnalia.com](mailto:cristina.elizetxea@tecnalia.com) (C.E.)

^3^ Maier Technology Centre, R&D Department, Polígono industrial Arabieta, Spain; [marord@maier.es](mailto:marord@maier.es) (M.O.)

***** Correspondence: [zvuluga@yahoo.com](mailto:zvuluga@yahoo.com) (Z.V.); [mcorobea@yahoo.com](mailto:mcorobea@yahoo.com) (M.C.C.); Tel.: +40-21-316-3068

**Table S1.** Formulations and processing conditions of all prepared and characterized samples

| **Sample** | **Components (wt.%)** | | | **Processing conditions** |
| --- | --- | --- | --- | --- |
|  | **PMMA** | **HNT** | **EBS** |  |
| *HNT modified with EBS* | | | | |
| (HNT-EBS)80 | - | 70 | 30 | 80 °C, 100 rpm, 1h |
| (HNT-EBS)120 | - | 70 | 30 | 120 °C, 100 rpm, 1h |
| (HNT-EBS)160 | - | 70 | 30 | 160 °C, 100 rpm, 1h |
| *PMMA based masterbatches* | | | | |
| CPA-80 | 80 | 14 | 6 | 240 °C (at die), 180 rpm |
| CPA-120 | 80 | 14 | 6 | 240 °C (at die), 180 rpm |
| CPA-160 | 80 | 14 | 6 | 240 °C (at die), 180 rpm |
| C-HNT | 80 | 20 | - | 240 °C (at die), 180 rpm |
| *PMMA/HNT nanocomposites* | | | | |
| PMMA-(HNT-EBS)80 | 97.14 | 2 | 0.86 | 240 °C (at die), 180 rpm |
| PMMA-(HNT-EBS)120 | 97.14 | 2 | 0.86 | 240 °C (at die), 180 rpm |
| PMMA-(HNT-EBS)160 | 97.14 | 2 | 0.86 | 240 °C (at die), 180 rpm |
| PMMA-HNT | 98 | 2 | - | 240 °C (at die), 180 rpm |
| PMMA-EBS | 99.14 | - | 0.86 | 240 °C (at die), 180 rpm |
| PMMA | 100 | - | - | 240 °C (at die), 180 rpm |

**(a)**

**(b)**

**Scheme S1.** Thermal decomposition of PMMA: **(a)** homolytic bond dissociation and **(b)** char production.

**Scheme S2.** Reactions suggested for thermal degradation of EBS – scission in the β position of the carbonyl group (able to generate species to react with PMMA degradation species).

| **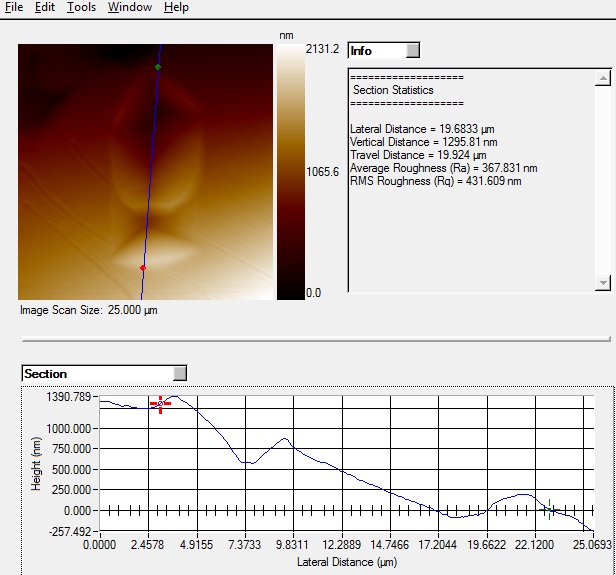**  **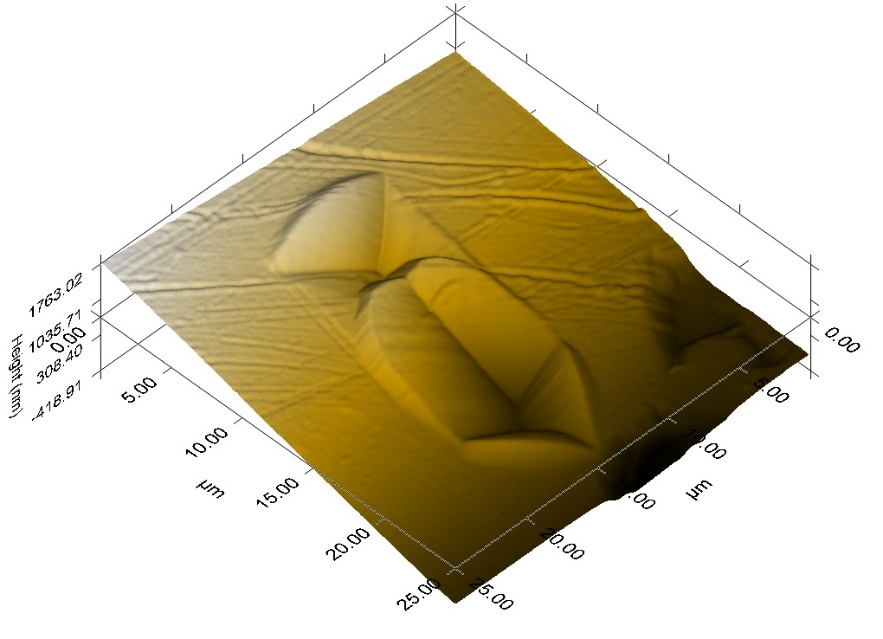** | 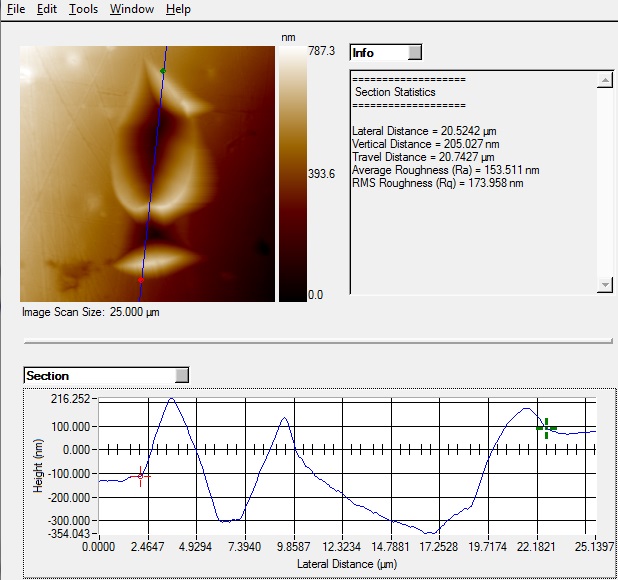  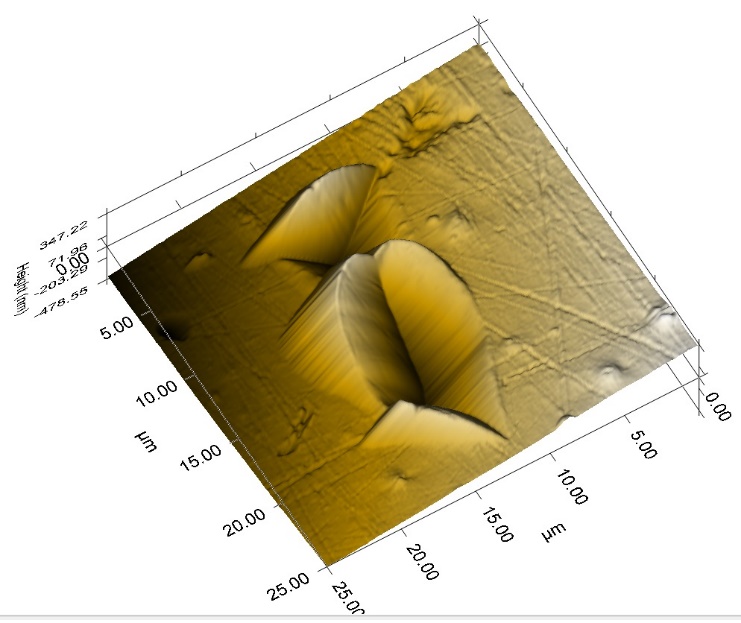 | 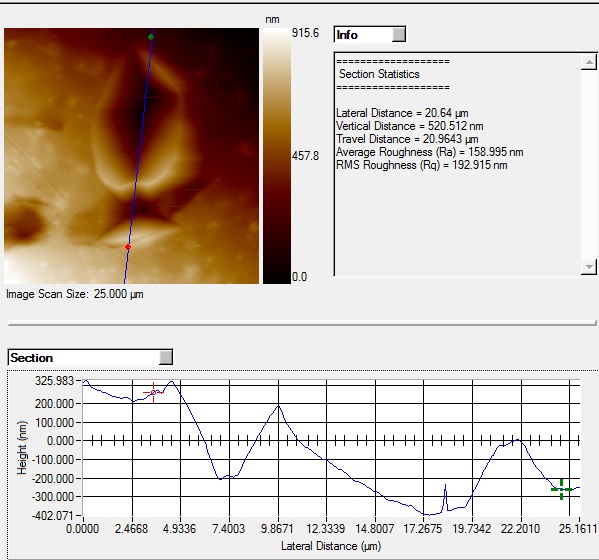  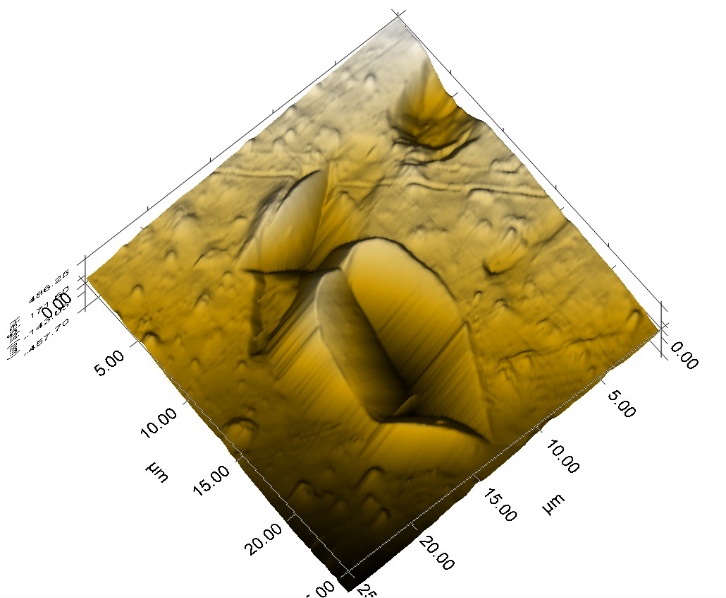 |
| --- | --- | --- |
| (**a**) | (**b**) | **(c)** |

**Figure S1.** 2-D and 3-D topographical in-situ SPM images obtained on: (**a**) PMMA; (**b**) PMMA-EBS; (**c**) PMMA-HNT.

| 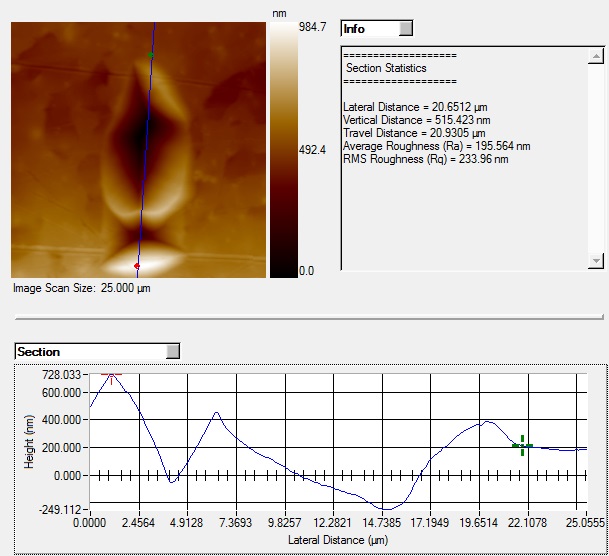  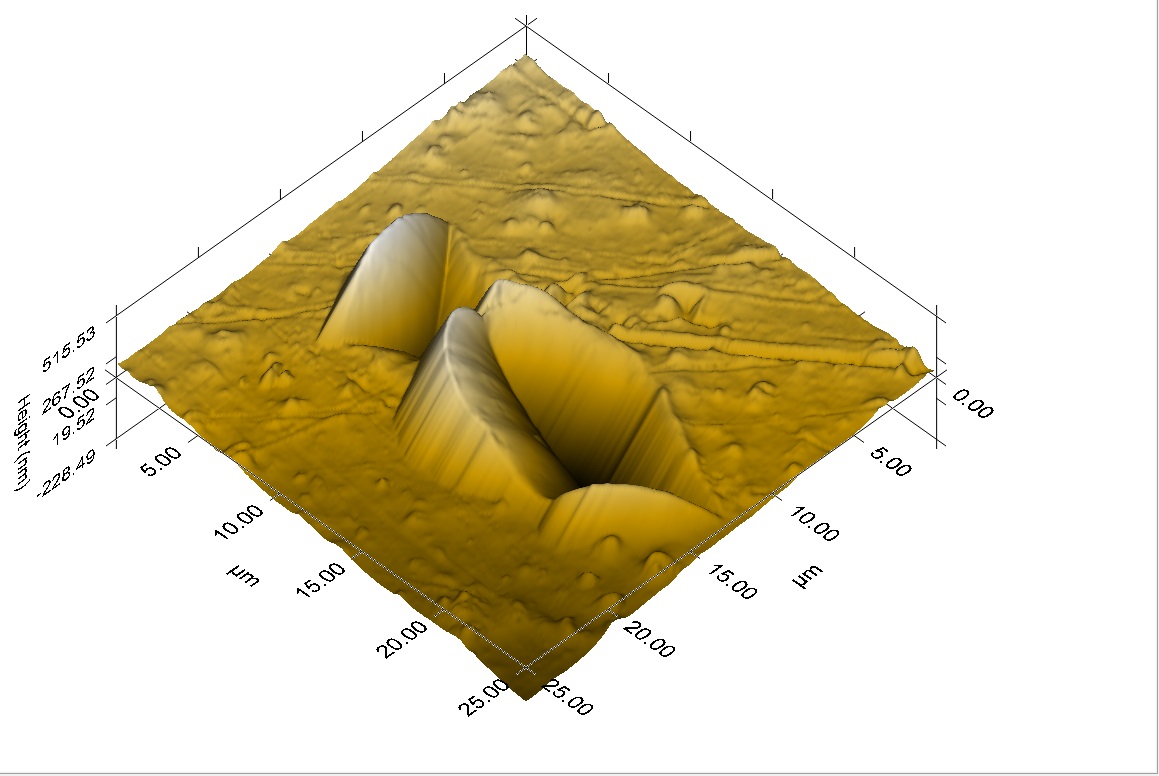 | 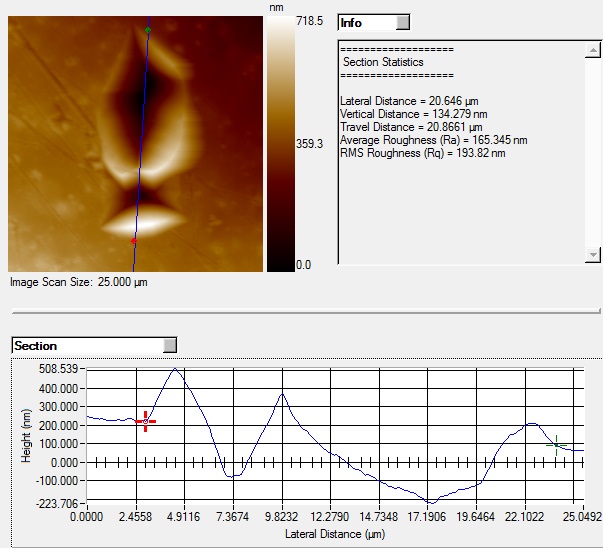  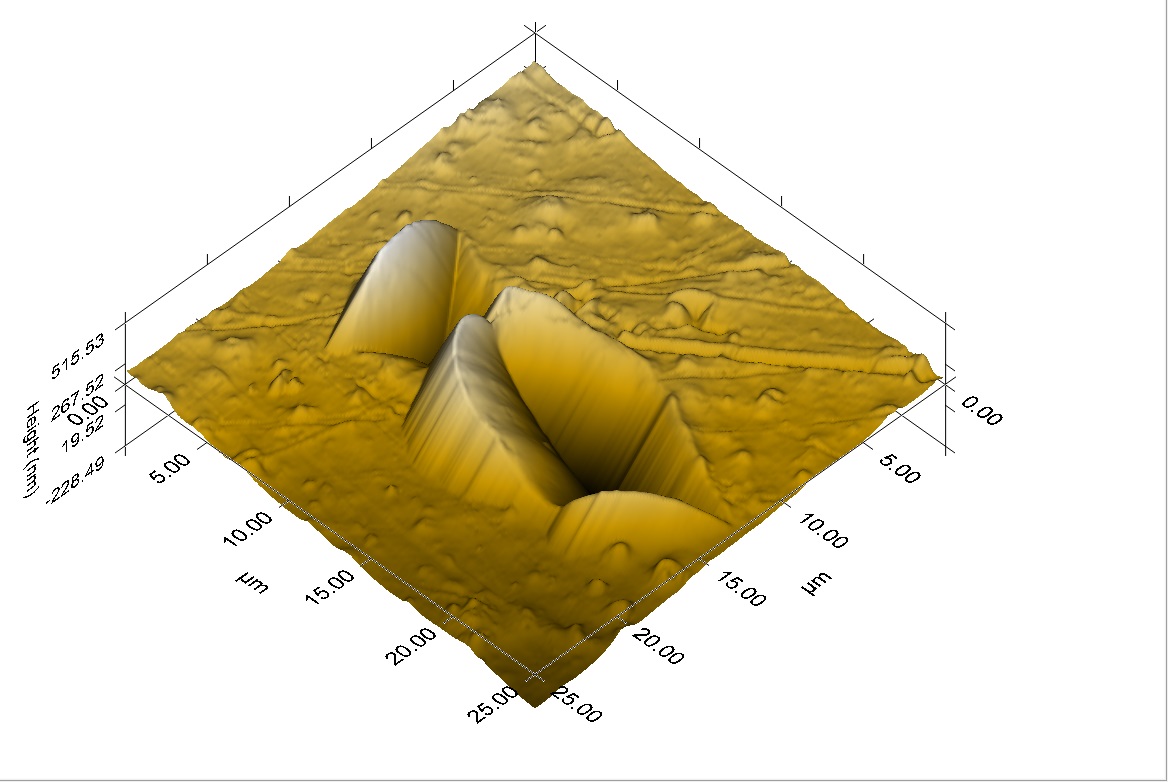 | 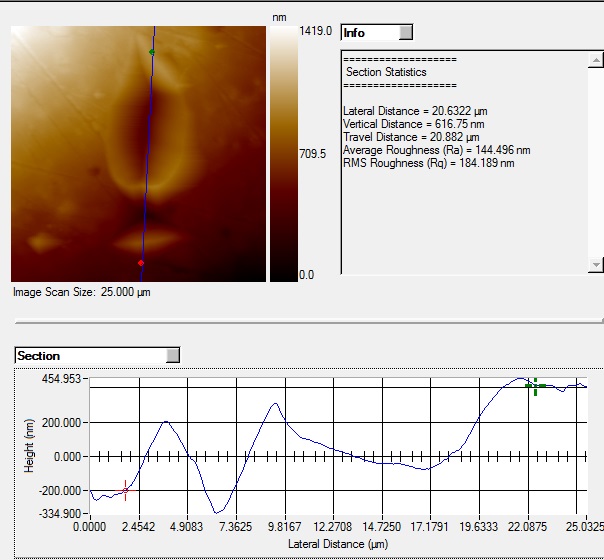  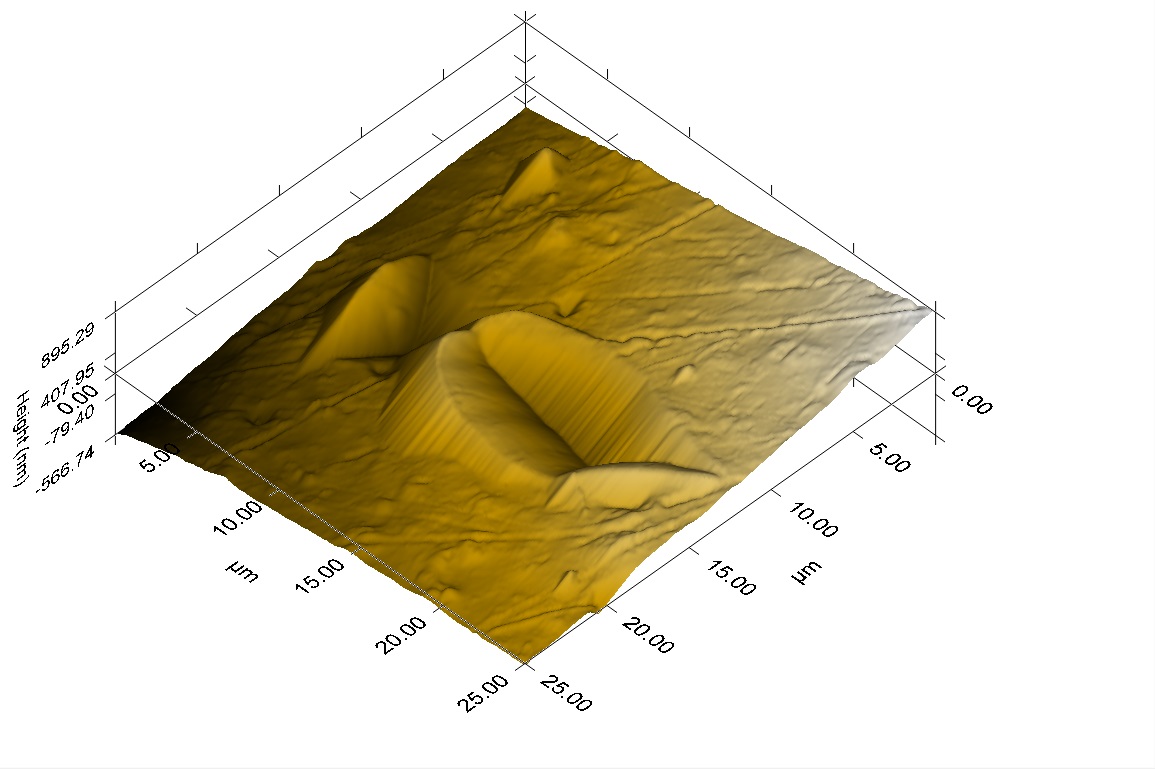 |
| --- | --- | --- |
| (**a**) | (**b**) | **(c)** |

**Figure S2.** 2-D and 3-D topographical in-situ SPM images obtained on: (**a**) PMMA-(HNT-EBS)80; (**b**) PMMA-(HNT-EBS)120; (**c**) PMMA-(HNT-EBS)160.
